# TARGET: A Randomized, Noninferiority Trial of a Pretest, Patient-Driven Genetic Education Webtool Versus Genetic Counseling for Prostate Cancer Germline Testing

**DOI:** 10.1200/PO.23.00552

**Published:** 2024-03-07

**Authors:** Stacy Loeb, Scott W. Keith, Heather H. Cheng, Amy E. Leader, Laura Gross, Tatiana Sanchez Nolasco, Nataliya Byrne, Rebecca Hartman, Lauren H. Brown, Christopher Michael Pieczonka, Leonard G. Gomella, William Kevin Kelly, Costas D. Lallas, Nathan Handley, Patrick Johnston Mille, James Ryan Mark, Gordon Andrew Brown, Sameer Chopra, Alexandra McClellan, David R. Wise, Lucas Hollifield, Veda N. Giri

**Affiliations:** ^1^ Department of Urology, NYU Langone Health, New York, NY; ^2^ Department of Population Health, NYU Langone Health, New York, NY; ^3^ Perlmutter Cancer Center, NYU Langone Health, New York, NY; ^4^ Department of Surgery/Urology, Manhattan Veterans Affairs, New York, NY; ^5^ Division of Biostatistics and Bioinformatics, Department of Pharmacology, Physiology and Cancer Biology, Thomas Jefferson University, Philadelphia, PA; ^6^ Division of Hematology and Oncology, Department of Medicine, University of Washington, Seattle, WA; ^7^ Fred Hutchinson Cancer Center, Seattle, WA; ^8^ Department of Medical Oncology, Thomas Jefferson University, Philadelphia, PA; ^9^ Yale Cancer Center, New Haven, CT; ^10^ Yale New Haven Health, New Haven, CT; ^11^ Associated Medical Professionals of NY, Syracuse, NY; ^12^ Department of Urology, Sidney Kimmel Cancer Center, Thomas Jefferson University, Philadelphia, PA; ^13^ Department of Integrative Medicine and Nutritional Sciences, Thomas Jefferson University, Philadelphia, PA; ^14^ New Jersey Urology, Summit Health, Voorhees, NJ; ^15^ Department of Medicine, Yale School of Medicine, New Haven, CT

## Abstract

**PURPOSE:**

Germline genetic testing (GT) is important for prostate cancer (PCA) management, clinical trial eligibility, and hereditary cancer risk. However, GT is underutilized and there is a shortage of genetic counselors. To address these gaps, a patient-driven, pretest genetic education webtool was designed and studied compared with traditional genetic counseling (GC) to inform strategies for expanding access to genetic services.

**METHODS:**

Technology-enhanced acceleration of germline evaluation for therapy (TARGET) was a multicenter, noninferiority, randomized trial (ClinicalTrials.gov identifier: NCT04447703) comparing a nine-module patient-driven genetic education webtool versus pretest GC. Participants completed surveys measuring decisional conflict, satisfaction, and attitudes toward GT at baseline, after pretest education/counseling, and after GT result disclosure. The primary end point was noninferiority in reducing decisional conflict between webtool and GC using the validated Decisional Conflict Scale. Mixed-effects regression modeling was used to compare decisional conflict between groups. Participants opting for GT received a 51-gene panel, with results delivered to participants and their providers.

**RESULTS:**

The analytic data set includes primary outcome data from 315 participants (GC [n = 162] and webtool [n = 153]). Mean difference in decisional conflict score changes between groups was –0.04 (one-sided 95% CI, –∞ to 2.54; *P* = .01), suggesting the patient-driven webtool was noninferior to GC. Overall, 145 (89.5%) GC and 120 (78.4%) in the webtool arm underwent GT, with pathogenic variants in 15.8% (8.7% in PCA genes). Satisfaction did not differ significantly between arms; knowledge of cancer genetics was higher but attitudes toward GT were less favorable in the webtool arm.

**CONCLUSION:**

The results of the TARGET study support the use of patient-driven digital webtools for expanding access to pretest genetic education for PCA GT. Further studies to optimize patient experience and evaluate them in diverse patient populations are warranted.

## Teaser

TARGET: A randomized, noninferiority trial of a pretest, patient-driven genetic education webtool vs. genetic counseling for #prostatecancer germline testing by @LoebStacy @vedangiri et al.

## INTRODUCTION

Approximately 5%-12% of patients with prostate cancer (PCA) harbor a germline pathogenic variant (PV).^1^ Early identification of these PVs allows for personalized PCA management. Previous studies have observed a greater risk of PCA upgrading during active surveillance and increased PCA lethality for those with germline PVs in *ATM*, *BRCA1*, or *BRCA2*.^2,3^ For patients with metastatic PCA, having germline PVs in DNA repair/homologous recombination repair genes, such as *BRCA1*, *BRCA2*, *ATM*, *or PALB2*, are indications for PARP inhibitor therapy because of reported clinical responses.^4-9^ Indeed, recognizing germline PV status in patients with PCA can not only inform their treatment, but also clinical trial eligibility, hereditary risk for other cancers, and cascade testing for at-risk relatives.^10,11^

**CONTEXT**

**Key Objective**
To compare pretest genetic education via a webtool for noninferiority to traditional genetic counseling (GC) in patients with prostate cancer (PCA).
**Knowledge Generated**
For the primary end point of decisional conflict, pretest counseling via a webtool was noninferior to traditional GC. Webtool participants had more negative attitudes toward testing after education and were less likely to opt for genetic testing.
**Relevance**
Genetic evaluation is underutilized in PCA and digital webtools may help expand access to pretest informed decision-making for germline testing.

Given these growing clinical implications, expert panels now recommend germline genetic testing (GT) for those with PCA that is metastatic, very high-risk, or high-risk; with Ashkenazi Jewish ancestry; or with additional personal or family history of cancer suspicious for known hereditary cancer syndromes.^9,12,13^ With more patients eligible for GT, demand for genetic services is rising.

Genetic counselors have traditionally provided pretest education, obtained informed consent, and facilitated germline GT in the oncology setting. Despite the growth of the genetic counselor workforce, and although many genetic counselors offer care through multiple service delivery models (eg, in-person, video, and telephone), lack of timely access to genetic counselors is an ongoing obstacle.^14,15^ Furthermore, existing genetic services remain unevenly distributed across the country, and social determinants of health such as household income further contribute to inequitable care.^16,17^ Mitigating such barriers may result in better outcomes for patients with PCA. Nongenetic providers are increasingly ordering GT, which sets the stage for tools to be used to help provide pretest information to patients.^15^

To address existing care gaps among patients with PCA, we designed a webtool to provide web-based genetic education (WBGE) via a patient-driven webtool, and conducted a randomized noninferiority trial to compare it with traditional pretest GC.

## METHODS

### Study Design

The technology-enhanced acceleration of germline evaluation for therapy (TARGET) study was a multicenter randomized controlled trial comparing WBGE versus traditional pretest GC. The study is registered (ClinicalTrials.gov identifier: NCT04447703), and a study protocol was previously published.^18^ Recruitment sites included Thomas Jefferson University, NYU Langone Health, Manhattan Veterans Affairs, University of Washington Medical Center, Fred Hutchinson Cancer Center, and Associated Medical Professionals of NY. In brief, we recruited US adults diagnosed with PCA eligible for genetic evaluation on the basis of guidelines because of disease characteristics (metastatic, prostate-specific antigen >20, grade group 4 or higher, stage ≥T3a, intraductal or cribriform histology, and biochemical recurrence), Ashkenazi Jewish ancestry, or family history criteria.^9,12^ Patients with previous germline GT for inherited cancer risk, cognitive impairment precluding informed consent, or who were non–English-speaking were excluded. The study was not limited to newly diagnosed patients; therefore, any patients without previous germline testing who otherwise met criteria were eligible. The study was approved by the institutional review board and informed consent was obtained from each participant.

Eligible participants underwent informed consent and were randomly assigned in a 1:1 fashion between the two arms: (1) traditional GC: meet with genetic counselor in-person or via telehealth for standard pretest counseling; or (2) WBGE: receive pretest education through a 9-module patient-driven webtool created by the study team and Prostate Cancer Foundation (22 minutes in total), with an individual login for tracking purposes. Each module of the webtool had a short quiz at the end to ensure understanding of the information. In both arms, the key elements of pretest genetic education included basics of cancer genetics and inheritance, genetic basis of PCA, risk factors for PCA, options for germline testing, types of test results (positive, negative, and variant of uncertain significance [VUS]), genes to be tested, benefits/risks/limitations of testing, management on the basis of test results, genetic discrimination laws, and implications for relatives.

Crossovers occurred if participants assigned to group 1 (GC) were unable to find a possible appointment time for the GC visit; for participants assigned to group 2 (WBGE), crossovers occurred if they were unable to successfully complete the knowledge questions at the end of each webtool module, or had unanswered questions that they wished to speak to a genetic counselor before deciding about GT.

In both arms, after GC/WBGE completion, participants were offered no-charge GT through Invitae with a custom 51-gene panel (including *ABRAXAS1*, *APC*, *ATM*, *AXIN2*, *BAP1*, *BARD1*, *BLM*, *BMPR1A*, *BRCA1*, *BRCA2*, *BRIP1*, *CDK4*, *CDKN2A*, *CHEK2*, *EPCAM*, *FANCA*, *FANCB*, *FANCC*, *FANCD2*, *FANCE*, *FANCF*, *FANCG*, *FANCI*, *FANCL*, *FANCM*, *GREM1*, *HOXB13*, *MLH1*, *MLH3*, *MRE11*, *MSH2*, *MSH6*, *MUTYH*, *NBN*, *PALB2*, *PMS2*, *POLD1*, *POLE*, *PTEN*, *RAD50*, *RAD51C*, *RAD51D*, *SMAD4*, *SMARCA4*, *SMARCB1*, *SMARCE1*, *STK11*, *TP53*, *WRN*, *WT1*, and *XRCC2*). A sample collection kit was sent to participants' homes for saliva collection to mail to the genetic laboratory for testing (by the genetic counselor for the GC arm and by the study coordinator for the WBGE arm). Clinical reports were sent to the ordering provider and discussed with participants by the research coordinator and/or genetic counselor.

Participants completed questionnaires online via REDCap; pertinent to the primary and secondary outcomes, surveys were taken at baseline (T1) and after the intervention (GC or WBGE; T2). The primary outcome was decisional conflict using the 16-item O'Connor Decisional Conflict Scale.^19^ Secondary outcomes included cancer genetics knowledge (adapted from Erblich et al^20^), attitudes toward GT,^21^ and satisfaction with GC (adapted from the Satisfaction with Genetic Counseling Scale).^22^ The GAD-7 scale^23^ was also used to capture baseline anxiety and any change for adverse event reporting (a change in GAD-7 score from T1 to T2 >4 related to the study).

Recruitment began in 2020 after institutional review board approval at the study sites. The study was powered on the basis of previous published studies^24^ using a noninferiority margin of four points for decisional conflict scores reflecting tolerance for a difference without indicating true inferiority. On the basis of previous studies,^24^ we estimated that the in-person pretest counseling mean decisional conflict in our target population to be approximately 6.7 with a standard deviation (SD) of 13.3. A sample size of n = 138 patients per group was necessary, under these assumptions, to have 80% power to detect noninferiority of WBGE to traditional GC by a one-sided t-test with 5% type I error rate. To account for loss to follow-up after random assignment of up to 20%, the study recruited n = 174 GC and n = 172 WBGE per group (n = 346 in total). The final analytic data set included primary outcome data from 315 participants (GC [n = 162] and webtool [n = 153]).

### Statistical Methods

Demographics and other descriptive data were summarized by frequency counts with percentages or means with SDs over the entire cohort and between study groups. A mixed-effects regression modeling approach with random intercepts was used to estimate the treatment effects of the WBGE tool versus traditional GC according to the modified intention-to-treat (mITT) principle. The pre-post changes on study end point scores were compared by fitting these models to include fixed effects for study group indicator (WBGE *v* traditional GC), time indicator (post-T2 [after intervention] *v* pre-T1 [at baseline]), their interaction (ie, the treatment effect of WBGE minus traditional GC), and a covariate to adjust for the study site (used to stratify the random assignment and to improve precision in the models). The primary end point was noninferiority for changes from T1 to T2 in decisional conflict of WBGE compared with traditional GC by a prespecified margin of δ = 4, which reflected our tolerance for a small, possibly existing difference still consistent with noninferior effect. We tested for noninferiority using a one-sided 95% CI and *P* value for the difference. All other end points were evaluated for treatment effects and two-sided 95% CIs by mixed-effects regression (mean adjusted differences in knowledge score and attitude score changes), linear regression (adjusted mean differences in satisfaction scores at T2), or modified Poisson regression^25^ (adjusted relative risk [RR] for GT uptake in WBGE relative to traditional GC).

**FIG 1.**
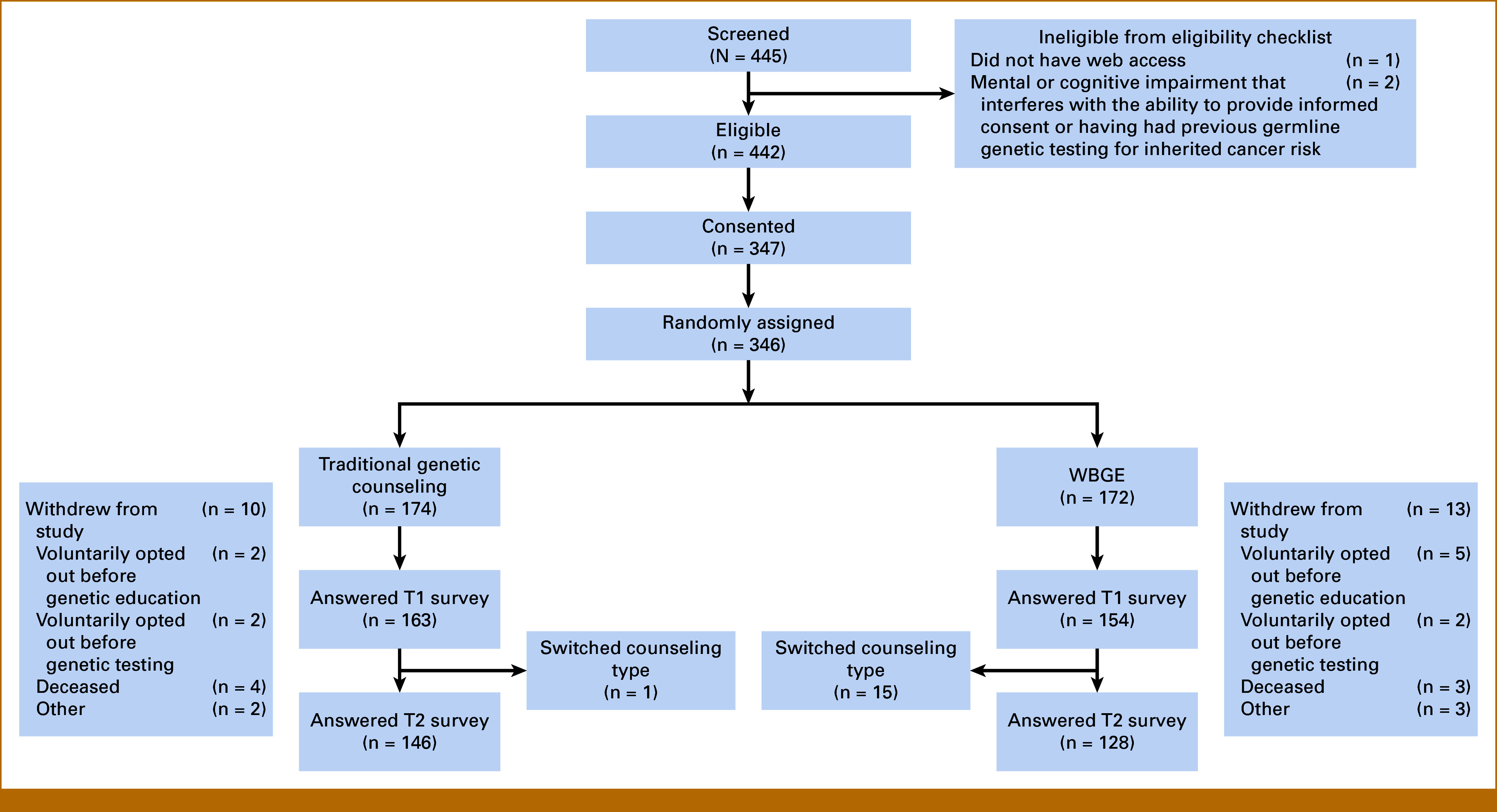
TARGET study CONSORT diagram. n = 315 people who answered the T1 survey had primary outcome data. TARGET, technology-enhanced acceleration of germline evaluation for therapy; WBGE, web-based genetic education via patient-driven webtool.

The analyses presented focus on those participants completing at least the T1 decisional conflict questionnaire. Figure 1 provides a summary of study patient flow, exclusions, dropouts, and sample sizes. We provide the results of sensitivity to participant crossovers who engaged in both arms per protocol and sensitivity to missing primary outcome data in the supplemental materials. Primary and secondary end point analyses were repeated on study data sets after excluding crossovers, placing crossovers in a separate study group, placing all the crossovers in the traditional GC group, or placing all the crossovers in the WBGE group (Appendix Table A1). Multiple regression imputation was used to impute missing data consistent with the null hypothesis of inferiority of WBGE to traditional GC by replacing missing decisional conflict scores with expected values among those assigned to the traditional GC group and with expected values plus the δ = 4 noninferiority margin for missing decisional conflict scores among those assigned to the WBGE group and repeated the primary end point analyses on these data with imputations (Appendix Tables A2 and A3).^24,26^ All statistical analyses were conducted using SAS v9.4 (SAS Institute, Inc, Cary, NC).

## RESULTS

Figure 1 shows the study flow. Of 445 patients screened, three were ineligible on the basis of not having web access (n = 1); and mental or cognitive impairment that interferes with the ability to provide informed consent or previous germline GT for inherited cancer risk (n = 2). Three hundred and forty-seven participants were consented and 346 were randomly assigned. Demographic and other descriptive data on the analytical sample (n = 315) with complete data on decisional conflict (primary end point) at the T1 time point are shown in Table 1. The mean age was 63.8 years at PCA diagnosis, 89.5% self-identified as White, and 2.9% identified as Hispanic/Latino, 16.8% reported Ashkenazi Jewish ancestry, and 77.1% had at least one first- or second-degree relative with prostate, breast, ovarian, colorectal, uterine, or pancreatic cancer. Demographics and baseline characteristics were reasonably well balanced between the 162 participants assigned to traditional GC and the 153 participants assigned to the WBGE groups, although 16 participants received both the WBGE and traditional GC.

**TABLE 1.** Demographics and Patient Characteristics Overall and Between Study Groups

Variable	Overall (N = 315)	Traditional GC (n = 162)	WBGE (n = 153)
Race, No. (%)			
White or Caucasian	282 (89.5)	144 (88.9)	138 (90.2)
Black or African American	23 (7.3)	12 (7.4)	11 (7.2)
Asian	6 (1.9)	5 (3.1)	1 (0.7)
American Indian/Alaska Native	1 (0.3)	0 (0)	1 (0.7)
Native Hawaiian/Pacific Islander	0 (0)	0 (0)	0 (0)
Other	3 (1)	1 (0.6)	2 (1.3)
Ethnicity, No. (%)			
Hispanic or Latino	9 (2.9)	2 (1.2)	7 (4.6)
Not Hispanic or Latino	304 (96.5)	160 (98.8)	144 (94.1)
Not sure	2 (0.6)	0 (0)	2 (1.3)
Ashkenazi Jewish ancestry, No. (%)			
Yes	53 (16.8)	31 (19.1)	22 (14.4)
No	250 (79.4)	124 (76.5)	126 (82.4)
Not sure	12 (3.8)	7 (4.3)	5 (3.3)
Education, No. (%)			
Less than college	39 (12.4)	19 (11.7)	20 (13.1)
College or more	276 (87.6)	143 (88.3)	133 (86.9)
Marital status, No. (%)			
Married/living with partner	247 (78.4)	125 (77.2)	122 (79.7)
Never married/separated/divorced/widowed	68 (21.6)	37 (22.8)	31 (20.3)
BMI (kg/m^2^)			
Mean (SD)	28.6 (5.4)	28.6 (5.4)	28.5 (5.5)
How servings of alcohol do you drink? No. (%)			
None	109 (34.6)	59 (36.4)	50 (32.7)
One to five per week	138 (43.8)	74 (45.7)	64 (41.8)
Six or more per week	68 (21.6)	29 (17.9)	39 (25.5)
Do you exercise regularly? No. (%)			
Yes	204 (64.8)	106 (65.4)	98 (64.1)
No	111 (35.2)	56 (34.6)	55 (36)
Age at PCa Dx, years			
Mean (SD)	63.8 (8.4)	64.3 (8.8)	63.3 (8)
Gleason score, No. (%)			
Less than or equal to 6	53 (16.8)	25 (15.4)	28 (18.3)
7 (3 + 4)	55 (17.5)	21 (13)	34 (22.2)
7 (4 + 3)	47 (14.9)	24 (14.8)	23 (15)
8-10	95 (30.2)	55 (34)	40 (26.1)
Don't know	57 (18.1)	35 (21.6)	22 (14.4)
What is the current stage of your PCA? No. (%)			
Only within the prostate	131 (41.6)	66 (40.7)	65 (42.5)
Spread to the seminal vesicles	37 (11.7)	17 (10.5)	20 (13.1)
Spread to lymph nodes	38 (12.1)	17 (10.5)	21 (13.7)
Distant spread (eg, to bones)	46 (14.6)	25 (15.4)	21 (13.7)
Don't know	58 (18.4)	34 (21)	24 (15.7)
What is the current status of your PCA? No. (%)			
No evidence of disease (cure)	74 (23.5)	35 (21.6)	39 (25.5)
Stable disease	67 (21.3)	31 (19.1)	36 (23.5)
Rising PSA but no findings on scans	36 (11.4)	22 (13.6)	14 (9.2)
Recurrence of disease	36 (11.4)	16 (9.9)	20 (13.1)
Unsure	94 (29.8)	54 (33.3)	40 (26.1)
Health literacy: range of possible scores is 1-10. Higher scores indicate greater literacy	8.64 (1.40)	8.82 (1.34)	8.44 (1.45)
Numeracy: percent of questions answered correctly	75.34% (23.09%)	75.31% (22.77%)	75.38% (23.50%)
Family history: any first-/second-degree relative who had a diagnosis of prostate, breast, ovarian, colorectal, uterine, or pancreatic cancer, No. (%)			
Yes	243 (77.1)	130 (80.2)	113 (73.9)
No	54 (17.1)	22 (13.6)	32 (20.9)

NOTE. Percentages are column percentages unless noted otherwise. Some participants were missing data for Gleason score (n = 8), current stage of PCA (n = 5), current status of PCA (n = 8), and family history (n = 18).

Abbreviations: GC, genetic counseling; PCA, prostate cancer; PSA, prostate-specific antigen; SD, standard deviation; WBGE, web-based genetic education via patient-driven webtool.

**FIG 2.**
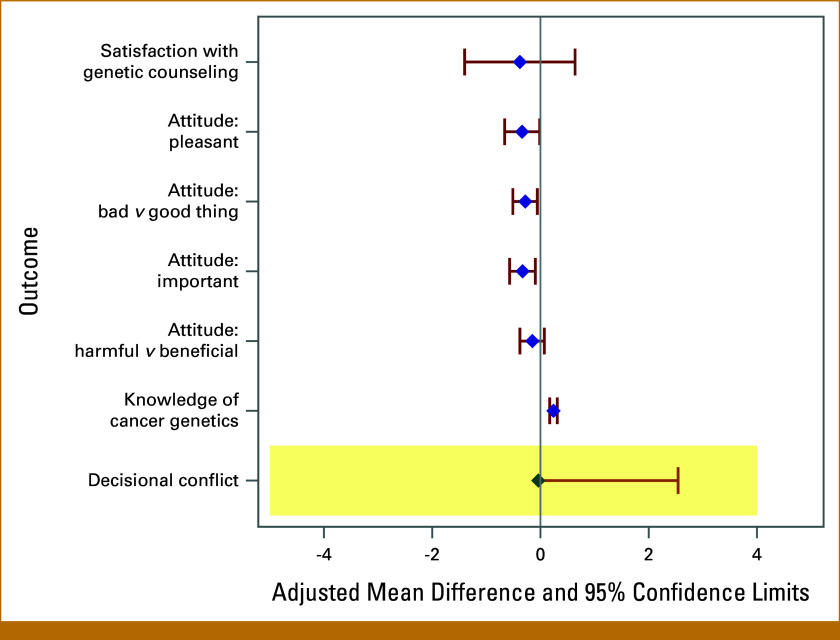
Study-adjusted mean difference effect estimates with 95% confidence limits. Estimates represent the mean difference between study groups WBGE (patient-driven webtool) minus traditional genetic counseling treatment groups in terms of change in scores (T2 minus T1), except for satisfaction scores, which were analyzed only at T2. The shaded region corresponds to the noninferiority rejection region for the primary end point, decisional conflict (–∞ to 4). WBGE, web-based genetic education via patient-driven webtool.

Figure 2 and Table 2 show mixed effects and linear regression modeling results for the primary and secondary end points. For the primary end point, mean difference in decisional conflict score changes between groups was –0.04 (one-sided 95% CI, –∞ to 2.54; *P* = .01), suggesting that WBGE with the patient-driven webtool was noninferior to traditional GC by the prespecified δ = 4. For secondary end points, a significant 24.1% greater improvement in knowledge scores was associated with WBGE versus traditional GC (95% CI, 17.1 to 31.1). We did not detect adjusted mean differences for satisfaction with GC or for changes in the GT attitude scores regarding harm versus benefit. However, the adjusted mean differences for changes in attitude scores for the importance (–0.33; 95% CI, –0.57 to –0.09), bad versus good (–0.28; 95% CI, –0.51 to –0.06), and pleasantness (–0.34; 95% CI, –0.66 to –0.01) of GT were significantly lower for WBGE compared with traditional GC.

**TABLE 2.** Primary and Secondary End Point Results

Score	Arm	Time	Adjusted Mean^a^	Mean Difference Between Groups^b^ (95% CI)
Decisional conflict	Traditional GC	T1	35.89	–0.04 (–∞ to 2.54)^c^
		T2	27.42	
		Change	–8.47	
	WBGE	T1	35.98	
		T2	27.46	
		Change	–8.51	
Knowledge of cancer genetics	Traditional GC	T1	32.4	24.1 (17.1 to 31.1)
		T2	66.5	
		Change	34.0	
	WBGE	T1	35.2	
		T2	93.3	
		Change	58.1	
Attitude: harmful *v* Beneficial	Traditional GC	T1	6.57	–0.15 (–0.38 to 0.07)
		T2	6.62	
		Change	0.045	
	WBGE	T1	6.49	
		T2	6.38	
		Change	–0.11	
Attitude: important	Traditional GC	T1	6.26	–0.33 (–0.57 to –0.09)
		T2	6.52	
		Change	0.25	
	WBGE	T1	6.39	
		T2	6.31	
		Change	–0.08	
Attitude: bad *v* good thing	Traditional GC	T1	6.45	–0.28 (–0.51 to –0.06)
		T2	6.64	
		Change	0.19	
	WBGE	T1	6.55	
		T2	6.46	
		Change	–0.09	
Attitude: pleasant	Traditional GC	T1	5.25	–0.34 (–0.66 to –0.01)
		T2	5.59	
		Change	0.34	
	WBGE	T1	5.36	
		T2	5.36	
		Change	0.005	
Satisfaction with genetic counseling	Traditional GC		27.67	–0.38 (–1.40 to 0.64)
	WBGE		27.29	

Abbreviations: GC, genetic counseling; WBGE, web-based genetic education via patient-driven webtool.

^a^ Mixed-effects regression modeled mean change in scores (T2 score–T1 score), adjusted for the study site that was used to stratify the random assignment.

^b^ Difference = WBGE change–traditional GC change, estimated by the group-by-time interaction term in the mixed-effects regression model.

^c^ One-sided CI for evaluating noninferiority of WBGE to traditional GC by the prespecified margin, δ = 4.

Uptake of GT among those randomly assigned was 89.5% in the traditional GC arm and 78.4% in the WBGE arm (RR = 0.88; 95% CI, 0.79 to 0.97). Of 265 participants with genetic results, PVs were identified overall in 42 participants (15.8%) and VUS in 87 participants (32.8%). When looking specifically at genes to test for PCA per National Comprehensive Cancer Network (*ATM*, *BRCA1*, *BRCA2*, *CHEK2*, *MSH6*, *PALB2*, and *HOXB13*), PVs were identified in 23 of 265 participants (8.7%).

Additional sensitivity analyses suggest that each of our main mITT effect estimates and 95% CIs presented above were robust to patient crossovers (Appendix Table A1) and to missing data on the primary end point at either T1 or T2 (Appendix Tables A2 and A3). A change in anxiety scores was identified in 7 (2.6%) of 274 participants who completed both the T1 and T2 surveys (Appendix Table A4), none of which were related to the study.

## DISCUSSION

Here, we report results of the first prospective randomized trial to our knowledge of a novel patient-driven webtool to deliver pretest genetic education for individuals with PCA. Given the expanding indications for germline testing and relative shortage of genetic counselors, studies such as these are needed to develop novel care pathways to facilitate pretest informed decision making for germline testing. In this study, we found that pretest genetic education via a patient-driven webtool was noninferior to traditional GC for the primary end point of decisional conflict to make an informed decision for GT. As making an informed decision for GT requires consideration of multiple components (such as information on cancer inheritance, types of genetic results, implications for patients and their families, and genetic discrimination laws), decisional conflict noninferiority for GT is important when evaluating the effectiveness of implementing similar digital pretest tools in practice. This is aligned with many previous studies that have assessed alternate delivery models for pretest genetic education on the basis of evaluation of decisional conflict for informed decision making for GT.^24,27,28^

Interestingly, in our study, participants in the WBGE arm also scored significantly better on genetic knowledge after the intervention, suggesting that the WBGE was a successful format to provide genetic education to patients with PCA. Furthermore, satisfaction with the genetic services was not significantly different whether participants received traditional GC or used the patient-driven webtool to make a decision for GT. Of interest, participants who used the webtool in the WBGE arm reported lower scores for the importance, value, and experience of GT, which deserves further study.

It is noteworthy that fewer participants in the WBGE arm underwent GT compared with the traditional GC arm. Similar findings have been reported in other studies using telemedicine/technological solutions for GC.^20^ For example, in a randomized trial of telephone versus in-person counseling for breast and ovarian cancer, telephone counseling was noninferior for the primary outcomes of knowledge, satisfaction, decision conflict, distress, and quality of life; however, *BRCA1/2* test uptake was lower in the telephone versus the in-person arms (84.2% *v* 90.1%).^24^ Reasons for this lower uptake of GT in the WBGE arm could include lower motivation for GT when not speaking to a genetic counselor, and the additional steps needed to collect and return their saliva specimen to the genetics laboratory. Some of the domains of attitude toward GT (eg, importance, bad *v* good, and pleasantness) were less favorable for participants in the WBGE arm, which may also have affected the ultimate uptake of GT. These factors and others need to be explored to optimize GT uptake when implementing future digital pretest tools.

Another interesting finding in our study was that PVs were identified in 15.1% of the study population, which is higher than some other published studies.^1,27,29^ This is particularly noteworthy considering the study population was mostly composed of individuals with locoregional prostate disease, who have a lower reported prevalence of germline PVs.^30^ This is likely related to the use of a more comprehensive 51-gene panel in our study, as several of these genes are not on standard PCA panels. More details on the germline variants that were identified, as well as downstream outcomes such as sharing results with families, will be reported separately.

Our study contributes to a growing body of literature on potential interventions to aid in the implementation of germline genetic services in practice. These results are important, given the substantial underutilization of germline testing among individuals with PCA. For example, in the US Health Information National Trends Survey, only 1% of patients with a history of PCA reported undergoing cancer-specific GT, compared with 52% of patients with breast/ovarian cancer (*P* = .001).^31^ This is concerning, given the substantial implications of germline genetic results for PCA screening and treatment, as well as informing hereditary cancer risk for patients and families.^10^

That notwithstanding, delivery of pretest genetic education is just one component of genetic services, and many other barriers must be overcome for optimal implementation of germline testing in practice. For example, in our previous qualitative work exploring the underutilization of germline testing among PCA health care providers, other barriers included variable knowledge and interest in GC and testing among patients, as well as variable knowledge of PCA genetics among nongenetics providers.^32^ We have previously worked on other technological innovations to address these barriers, including development and prospective evaluation of the Prostate Cancer Genetics Podcast to inform the public (available on podcasting platforms)^33^ and of the Helix application to provide education and decisional support for health care providers (available at helix.guide).^34^ The patient-driven webtool adds to this expanding armamentarium of digital applications to support broader awareness of and access to germline genetic services in PCA.

There are some limitations to note. Some participants did not complete final surveys, despite numerous attempts by the study team. However, extensive sensitivity analyses were conducted to address missing data and showed no impact on overall study findings. Another limitation is the limited racial and ethnic diversity among the study population. The underutilization of GT among Black and Hispanic patients with PCA must be addressed to reduce disparities in access to precision therapies, clinical trials, and knowledge of hereditary cancer risk. Furthermore, rates of VUS are notably higher in Black and Hispanic populations; many could be resolved with greater inclusion of minority and underrepresented populations in genetic studies.^35,36^ Additionally, the patient-driven webtool requires online access, which can pose a barrier to those without stable access to Wi-Fi, computers, etc, and raises concern about widening disparities. There are ongoing studies to examine ways to improve genetic education and uptake of GT in more diverse populations. Finally, implementation of the webtool in practice may be facilitated by having trained staff available to answer any patient questions that may arise.

In conclusion, these results support the use of a patient-driven digital webtool for expanding access to pretest education for germline GT among patients with PCA. Further studies are needed to explore enhancing patient experience, provider engagement, reducing disparities in genetics engagement, and expanding access to genetic services for patients with PCA across populations.

## DATA SHARING STATEMENT

The principal investigator will be primarily responsible for publication of study findings. Neither the complete nor any part of the results of the study carried out under this protocol will be published or passed on to any third party without the consent of the study principal investigator. Any investigator involved with this study is obligated to provide the principal investigator with complete results.

## APPENDIX

**TABLE A1.** Sensitivity to Crossovers in Primary and Secondary Outcomes Analysis Results

Outcome	Adjusted Mean Difference^a^ or RR^b^ Between Arms	95% CI
Decisional conflict		
mITT	–0.04	–∞ to 2.54
Exclude crossovers	0.19	–∞ to 2.78
Separate crossovers	0.18	–∞ to 2.86
Crossovers in traditional	0.28	–∞ to 2.90
Crossovers in WBGE	0.05	–∞ to 2.63
Satisfaction with genetic counseling		
mITT	–0.38	–1.40 to 0.64
Exclude crossovers	–0.33	–1.37 to 0.71
Separate crossovers	–0.33	–1.39 to 0.73
Crossovers in traditional	–0.28	–1.31 to 0.75
Crossovers in WBGE	–0.35	–1.37 to 0.67
Knowledge of cancer genetics		
mITT	24.1	17.06 to 31.14
Exclude crossovers	25.3	18 to 32.70
Separate crossovers	25.3	18 to 32.60
Crossovers in traditional	23.4	16.20 to 30.60
Crossovers in WBGE	24.5	17.50 to 31.50
Attitude: harmful *v* beneficial		
mITT	–0.15	–0.38 to 0.072
Exclude crossovers	–0.16	–0.40 to 0.08
Separate crossovers	–0.16	–0.39 to 0.075
Crossovers in traditional	–0.15	–0.38 to 0.08
Crossovers in WBGE	–0.15	–0.38 to 0.073
Attitude: important		
mITT	–0.33	–0.57 to –0.094
Exclude crossovers	–0.33	–0.58 to –0.087
Separate crossovers	–0.33	–0.58 to –0.087
Crossovers in traditional	–0.30	–0.54 to –0.061
Crossovers in WBGE	–0.33	–0.57 to –0.095
Attitude: bad *v* good thing		
mITT	–0.28	–0.51 to –0.058
Exclude crossovers	–0.29	–0.53 to –0.055
Separate crossovers	–0.29	–0.52 to –0.06
Crossovers in traditional	–0.27	–0.50 to –0.045
Crossovers in WBGE	–0.28	–0.51 to –0.059
Attitude: pleasant		
mITT	–0.34	–0.66 to –0.014
Exclude crossovers	–0.38	–0.72 to –0.047
Separate crossovers	–0.38	–0.72 to –0.048
Crossovers in traditional	–0.38	–0.71 to –0.054
Crossovers in WBGE	–0.34	–0.66 to –0.014
Genetic testing uptake (relative risk)		
mITT	0.87	0.79 to 0.96
Exclude crossovers	0.84	0.76 to 0.94
Separate crossovers	0.75	0.67 to 0.83
Crossovers in traditional	0.83	0.75 to 0.92
Crossovers in WBGE	0.87	0.79 to 0.96

Abbreviations: GC, genetic counseling; mITT, modified intention-to-treat; RR, relative risk; WBGE, web-based genetic education via patient-driven webtool.

^a^ Difference in scores = T2 score–T1 score.

^b^ RR = $\frac{\text{Probability}\;\text{of}\;\text{genetic}\;\text{testing}\;\text{in}\;\text{WBGE}\;}{\text{Probability}\;\text{of}\;\text{genetic}\;\text{testing}\;\text{in}\;\text{traditional}\;\text{GC}}$.

**TABLE A2.** Primary Outcome Missing Data Distribution Summary

Timepoint with Missing Data	Traditional Genetic Counseling (n = 174), No. (%)	WBGE (n = 172), No. (%)	Total (N = 346), No. (%)
Missing at T1 only	0 (0)	0 (0)	0 (0)
Missing at T2 only	17 (9.8)	25 (14.5)	42 (12.1)
Missing at both T1 and T2	12 (6.9)	19 (11.1)	31 (9)

Abbreviation: WBGE, web-based genetic education via patient-driven webtool.

**TABLE A3.** Sensitivity to Missing Data in Primary Outcomes Analysis Results

Decisional Conflict	Adjusted Mean Difference^a^ Between Arms	95% CI
mITT	–0.04	–∞ to 2.54
Imputation^b^	0.99	–∞ to 3.22
Imputation^b^ and crossovers in traditional	0.95	–∞ to 3.38

Abbreviations: GC, genetic counseling; mITT, modified intention-to-treat; PCA, prostate cancer; WBGE, web-based genetic education via patient-driven webtool.

^a^ Difference in scores = T2 score–T1 score.

^b^ Multiple regression imputation was used to replace missing data with expected values consistent with the null hypothesis of inferiority of WBGE to traditional GC. The predictors in the longitudinal mixed-effect regression models constructed to estimate the imputed decisional conflict values included study site, study group assignment, time indicator (T2 *v* T1), race, current PCA status, alcohol consumption (≥six drinks/wk *v* fewer), age at PCA diagnosis, BMI, and time × study group interaction.

**TABLE A4.** Frequency (%) of Adverse Events (a change in GAD-7 score from T1 to T2 survey >4) for Those Who Completed the T1 and T2 Survey (none were related to the study)

Anxiety	Overall (N = 274), No. (%)	Traditional GC (n = 146), No. (%)	WBGE (n = 128), No. (%)
Anxiety adverse event	7 (2.6)	2 (1.4)	5 (3.9)
No anxiety adverse event	266 (97.1)	143 (97.9)	123 (96.1)

NOTE. Data on anxiety were missing for one participant.

Abbreviations: GC, genetic counseling; WBGE, web-based genetic education via patient-driven webtool.

## References

[1] Pritchard CC, Mateo J, Walsh MF, et al: Inherited DNA-repair gene mutations in men with metastatic prostate cancer. N Engl J Med 375:443-453, 2016 27433846 10.1056/NEJMoa1603144 PMC4986616

[2] Na R, Zheng SL, Han M, et al: Germline mutations in ATM and BRCA1/2 distinguish risk for lethal and indolent prostate cancer and are associated with early age at death. Eur Urol 71:740-747, 2017 27989354 10.1016/j.eururo.2016.11.033 PMC5535082

[3] Carter HB, Helfand B, Mamawala M, et al: Germline mutations in ATM and BRCA1/2 are associated with grade reclassification in men on active surveillance for prostate cancer. Eur Urol 75:743-749, 2019 30309687 10.1016/j.eururo.2018.09.021 PMC6699614

[4] Giri VN, Hyatt C, Gomella LG: Germline testing for men with prostate cancer: Navigating an expanding new world of genetic evaluation for precision therapy and precision management. J Clin Oncol 37:1455-1459, 2019 30978156 10.1200/JCO.18.02181

[5] de Bono J, Mateo J, Fizazi K, et al: Olaparib for metastatic castration-resistant prostate cancer. N Engl J Med 382:2091-2102, 2020 32343890 10.1056/NEJMoa1911440

[6] Abida W, Patnaik A, Campbell D, et al: Rucaparib in men with metastatic castration-resistant prostate cancer harboring a BRCA1 or BRCA2 gene alteration. J Clin Oncol 38:3763-3772, 2020 32795228 10.1200/JCO.20.01035 PMC7655021

[7] Chi KN, Rathkopf D, Smith MR, et al: Niraparib and abiraterone acetate for metastatic castration-resistant prostate cancer. J Clin Oncol 41:3339-3351, 2023 36952634 10.1200/JCO.22.01649 PMC10431499

[8] Saad F, Clarke NW, Oya M, et al: Olaparib plus abiraterone versus placebo plus abiraterone in metastatic castration-resistant prostate cancer (PROpel): Final prespecified overall survival results of a randomised, double-blind, phase 3 trial. Lancet Oncol 24:1094-1108, 2023 37714168 10.1016/S1470-2045(23)00382-0

[9] National Comprehensive Cancer Network clinical practice guidelines in oncology. Prostate cancer version 1.2023. https://www.nccn.org/professionals/physician_gls/pdf/prostate.pdf 10.6004/jnccn.2023.0006 36791750

[10] Loeb S, Giri VN: Clinical implications of germline testing in newly diagnosed prostate cancer. Eur Urol Oncol 4:1-9, 2021 33390340 10.1016/j.euo.2020.11.011

[11] Carlo MI, Giri VN, Paller CJ, et al: Evolving intersection between inherited cancer genetics and therapeutic clinical trials in prostate cancer: A white paper from the Germline Genetics Working Group of the Prostate Cancer Clinical Trials Consortium. JCO Precis Oncol 10.1200/PO.18.00060 10.1200/PO.18.00060 PMC6370313 30761386

[12] National Comprehensive Cancer Network clinical practice guidelines in oncology (NCCN Guidelines®): Genetic/familial high-risk assessment: Breast, ovarian, and pancreatic (version 3.2023). NCCN.org 10.6004/jnccn.2021.0001 33406487

[13] Giri VN, Knudsen KE, Kelly WK, et al: Implementation of germline testing for prostate cancer: Philadelphia Prostate Cancer Consensus Conference 2019. J Clin Oncol 38:2798-2811, 2020 32516092 10.1200/JCO.20.00046 PMC7430215

[14] Hoskovec JM, Bennett RL, Carey ME, et al: Projecting the supply and demand for certified genetic counselors: A workforce study. J Genet Couns 27:16-20, 2018 29052810 10.1007/s10897-017-0158-8

[15] Paller CJ, Antonarakis ES, Beer TM, et al: Germline genetic testing in advanced prostate cancer; practices and barriers: Survey results from the Germline Genetics Working Group of the Prostate Cancer Clinical Trials Consortium. Clin Genitourin Cancer 17:275-282.e1, 2019 31171481 10.1016/j.clgc.2019.04.013 PMC6662206

[16] Stoll K, Kubendran S, Cohen SA: The past, present and future of service delivery in genetic counseling: Keeping up in the era of precision medicine. Am J Med Genet C Semin Med Genet 178:24-37, 2018 29512888 10.1002/ajmg.c.31602

[17] Bellaiche MMJ, Fan W, Walbert HJ, et al: Disparity in access to oncology precision care: A geospatial analysis of driving distances to genetic counselors in the U.S. Front Oncol 11:689927, 2021 34222017 10.3389/fonc.2021.689927 PMC8242948

[18] Loeb S, Cheng HH, Leader A, et al: Technology-enhanced AcceleRation of Germline Evaluation for Therapy (TARGET): A randomized controlled trial of a pretest patient-driven webtool vs. genetic counseling for prostate cancer germline testing. Contemp Clin Trials 119:106821, 2022 35710085 10.1016/j.cct.2022.106821

[19] O'Connor AM: Validation of a decisional conflict scale. Med Decis Making 15:25-30, 1995 7898294 10.1177/0272989X9501500105

[20] Erblich J, Brown K, Kim Y, et al: Development and validation of a Breast Cancer Genetic Counseling Knowledge Questionnaire. Patient Educ Couns 56:182-191, 2005 15653247 10.1016/j.pec.2004.02.007

[21] Marteau TM, Dormandy E, Michie S: A measure of informed choice. Health Expect 4:99-108, 2001 11359540 10.1046/j.1369-6513.2001.00140.x PMC5060053

[22] DeMarco TA, Peshkin BN, Mars BD, et al: Patient satisfaction with cancer genetic counseling: A psychometric analysis of the Genetic Counseling Satisfaction Scale. J Genet Couns 13:293-304, 2004 19736695 10.1023/b:jogc.0000035523.96133.bc PMC3551590

[23] Spitzer RL, Kroenke K, Williams JB, et al: A brief measure for assessing generalized anxiety disorder: The GAD-7. Arch Intern Med 166:1092-1097, 2006 16717171 10.1001/archinte.166.10.1092

[24] Schwartz MD, Valdimarsdottir HB, Peshkin BN, et al: Randomized noninferiority trial of telephone versus in-person genetic counseling for hereditary breast and ovarian cancer. J Clin Oncol 32:618-626, 2014 24449235 10.1200/JCO.2013.51.3226 PMC3927731

[25] Zou G: A modified Poisson regression approach to prospective studies with binary data. Am J Epidemiol 159:702-706, 2004 15033648 10.1093/aje/kwh090

[26] Koch GG: Comments on 'Current issues in non-inferiority trials' by Thomas R. Fleming, Statistics in Medicine, DOI: 10.1002/sim.2855. Stat Med 27:333-342, 2008 17530625 10.1002/sim.2923

[27] Kwon DH, Gordon KM, Tong B, et al: Implementation of a telehealth genetic testing station to deliver germline testing for men with prostate cancer. JCO Oncol Pract 19:e773-e783, 2023 36649492 10.1200/OP.22.00638

[28] McCuaig JM, Tone AA, Maganti M, et al: Modified panel-based genetic counseling for ovarian cancer susceptibility: A randomized non-inferiority study. Gynecol Oncol 153:108-115, 2019 30638766 10.1016/j.ygyno.2018.12.027

[29] Genetics of prostate cancer (PDQ®)—Health professional version. National Cancer Institute. www.cancer.gov

[30] Giri VN, Obeid E, Gross L, et al: Inherited mutations in men undergoing multigene panel testing for prostate cancer: Emerging implications for personalized prostate cancer genetic evaluation. JCO Precis Oncol 10.1200/PO.16.00039 10.1200/PO.16.00039 PMC8210976 34164591

[31] Thakker S, Loeb S, Giri VN, et al: Attitudes, perceptions, and use of cancer-based genetic testing among healthy U.S. adults and those with prostate or breast/ovarian cancer. Urol Pract 10:26-32, 2023 37103438 10.1097/UPJ.0000000000000352

[32] Loeb S, Li R, Sanchez Nolasco T, et al: Barriers and facilitators of germline genetic evaluation for prostate cancer. Prostate 81:754-764, 2021 34057231 10.1002/pros.24172

[33] Loeb S, Sanchez Nolasco T, Siu K, et al: Usefulness of podcasts to provide public education on prostate cancer genetics. Prostate Cancer Prostatic Dis 26:772-777, 2023 36681741 10.1038/s41391-023-00648-4

[34] Giri VN, Walker A, Gross L, et al: Helix: A digital tool to address provider needs for prostate cancer genetic testing in clinical practice. Clin Genitourin Cancer 20:e104-e113, 2022 35012874 10.1016/j.clgc.2021.11.009

[35] Kwon DH, Borno HT, Cheng HH, et al: Ethnic disparities among men with prostate cancer undergoing germline testing. Urol Oncol 38:80.e1-80.e7, 2020 10.1016/j.urolonc.2019.09.010 31630993

[36] Giri VN, Hartman R, Pritzlaff M, et al: Germline variant spectrum among African American men undergoing prostate cancer germline testing: Need for equity in genetic testing. JCO Precis Oncol 10.1200/PO.22.00234 10.1200/PO.22.00234 PMC9200399 35666082

